# First Evidence of Natural SARS-CoV-2 Infection in Domestic Rabbits

**DOI:** 10.3390/vetsci9020049

**Published:** 2022-01-27

**Authors:** Matthieu Fritz, Daphné de Riols de Fonclare, Déborah Garcia, Stéphanie Beurlet, Pierre Becquart, Serge G. Rosolen, Alexandra Briend-Marchal, Eric M. Leroy

**Affiliations:** 1 Maladies Infectieuses et Vecteurs, Ecologie, Génétique, Evolution et Contrôle (MIVEGEC), University Montpellier, IRD, CNRS, 34394 Montpellier, France; matthieu.fritz@ird.fr (M.F.); daphne.fonclare@gmail.com (D.d.R.d.F.); deborah.garcia@ird.fr (D.G.); pierre.becquart@ird.fr (P.B.); 2 Laboratoire de Biologie Vétérinaire VEBIO, 94110 Arcueil, France; stephanie.beurlet@gmail.com (S.B.); briendmarchal@gmail.com (A.B.-M.); 3 Institut de la Vision, Sorbonne Université, INSERM, CNRS, 75012 Paris, France; serge.rosolen@inserm.fr; 4 Clinique Vétérinaire Voltaire, 92600 Asnières, France

**Keywords:** SARS-CoV-2, COVID-19, rabbits, pet rabbit, France, Luminex, serology, microsphere immunoassay, one health

## Abstract

We tested 144 pet rabbits sampled in France between November 2020 and June 2021 for antibodies to severe acute respiratory syndrome coronavirus 2 (SARS-CoV-2) by microsphere immunoassay. We reported the first evidence of a natural SARS-CoV-2 infection in rabbits with a low observed seroprevalence between 0.7% and 1.4%.

## 1. Introduction

Since the late 2019 emergence of SARS-CoV-2 in China, many studies have described the high propensity of SARS-COV-2 to cross the species barrier and infect a diversity of wild and domesticated animal species by experimental or natural infection [1]. This propensity may facilitate the establishment of new zoonotic reservoirs of SARS-CoV-2. Such an event, recently suggested following the observation of SARS-CoV-2 infections in white-tailed deer [2,3,4], could become a significant public health issue, as the human population may be susceptible to the reintroduction of viral strains with unknown pathology that have evolved in the reservoir species.

Rabbits are animals that humans interact with as pets, livestock, and wildlife. Compared to other companion pets (primarily dogs and cats [5]), livestock (mink [6]), or wild animals (white-tailed deer [2]), rabbits have been little investigated [7,8,9]. Indeed, there has been only one study, which noted an absence of SARS-CoV-2-specific antibodies among 29 rabbits kept as pets in Poland [10]. To take a closer look at possible SARS-CoV-2 infections in pet rabbits, we determined seroprevalence from 144 blood samples collected in France between November 2020 to June 2021 by veterinarians during health care visits. These rabbits were kept as pets in households with unknown SARS-CoV-2 status.

## 2. Materials and Methods

### 2.1. Sampling

The 144 blood samples were collected in dry/EDTA tubes from rabbits at the veterinary clinics. After centrifugation, the serum/plasma was kept at +4 °C until it was sent to VEBIO, a veterinary diagnostic laboratory. Shipping was conducted under safe and rapid conditions to limit contamination and ensure samples reached VEBIO within 48 h. An aliquot was taken from the sample for biomedical analyses requested by the veterinarians, and another aliquot was stored at +4 °C until it was sent to the MIVEGEC lab, Montpellier, where serological analyses were carried out. For shipping to the MIVEGEC lab, all samples were placed in refrigerated coolers and transported by a specialized professional carrier to ensure optimal safety conditions. Finally, the samples were stored at the MIVEGEC lab at −20 °C until testing. Data (age, sex, anamnestic when available) from rabbits were provided totally anonymized by VEBIO to MIVEGEC lab.

### 2.2. Microsphere Immunoassay (MIA)

The 144 rabbit serum samples were tested using a multiplex microsphere immunoassay (MIA). Ten µg of two recombinant SARS-CoV-2 antigens, receptor-binding domain (RBD) and trimeric spike (tri-S) (The Native Antigen Company, Kidlington, UK), were used to capture specific serum antibodies. Distinct MagPlex microsphere sets (Luminex Corp, Austin, TX, USA) were respectively coupled to viral antigens using the amine coupling kit (Bio-Rad Laboratories, Marnes-la-Coquette, France) according to the manufacturer’s instructions. Microsphere mixtures were successively incubated with serum samples (1:400), biotinylated protein A and biotinylated protein G (4 µg/mL each) (Thermo Fisher Scientific, Illkirch, France), and streptavidin-R-phycoerythrin (4 µg/mL) (Life technologies, Illkirch, France) on an orbital shaker and protected from light. Measurements were performed using a Luminex 200 instrument (Luminex Corp, Austin, TX, USA), and at least 100 events were read for each bead set. Binding events were displayed as median fluorescence intensities (MFI). These two tests were previously successfully used for dogs and cats [11,12,13].

## 3. Results

We tested the 144 blood samples by microsphere assay using beads coupled with a spike receptor-binding domain (RBD) and trimeric spike (tri-S) antigens (see Section 2). The average age of rabbits was 4.4 years (range: 1 to 8), 41% were females, 53% were males, and for 6%, the information on sex was unavailable. In the absence of prepandemic samples, and based on the hypothesis of a low incidence of natural infections in domestic rabbits, we chose a cutoff for determining positivity as three standard deviations above the average signal for all samples.

**Figure 1:**
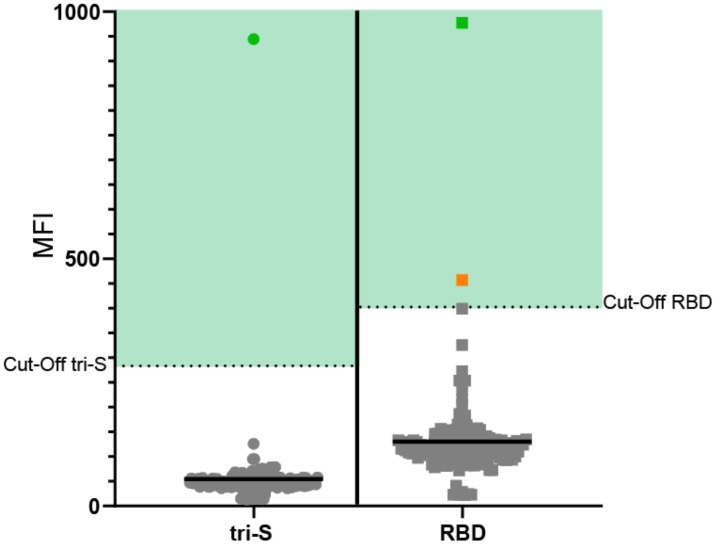
Serological evaluation of antiSARS-CoV-2 antibodies in pet rabbits in France. The green dots represent the rabbit positive for both antigens, whereas the orange dot represents the rabbit positive for RBD antigen only. The solid black line indicates the average fluorescent intensity. The same population was used to determine the cutoff for positivity (mean + 3× standard deviation).

Using this cutoff, we found one sample (female) seropositive for both antigens and another (female) positive only for RBD (Figure 1). For positive rabbits, at the time of sampling, no particular symptom consistent with SARS-CoV-2 infection was noted by the veterinarians. The double positive rabbit presented intermittent vestibular syndrome, which is common among rabbits.

Depending on the criteria used to determine positivity, we observed a seroprevalence of 0.7% using the most restrictive positivity criterion of positive results for two antigens, and 1.4% following a positive result for at least one antigen.

## 4. Discussion

To our knowledge, this is the first evidence of a natural SARS-CoV-2 infection in rabbits, most likely acquired from a COVID-19-positive owner. The sampling period of November 2020 to June 2021 includes peaks of the second and third epidemic waves of SARS-CoV-2 in France. Furthermore, although antibody detection alone does not mean productive infection with viral replication, our results are supported by previous studies showing active SARS-CoV-2 replication in rabbits after experimental infection without clinical signs of disease [8,9].

The very low observed seroprevalence could arise from a low susceptibility of rabbits to the virus and the generally more distant contact between pet rabbits and their owners. In experimental studies, productive infections with SARS-CoV-2 could only be established with a relatively high viral dose (10^5^ TCDI50), and led to low viral replication [8,9]. Another study failed to establish productive infections or detectable neutralizing antibody production in three captured wild cottontail rabbits experimentally infected by SARS-CoV-2 [7]. The contact rate of rabbits with the virus shed by their infected owners could also be reduced compared to dogs and cats, who have much more intimate contact with their owners, often licking their hands and face and even sleeping with them, which would favor transmission of the virus.

The very low seroprevalence we observed among the 144 pet rabbits sampled during a period, including the second and third human epidemic wave in France, indicates that infections of rabbits are rare and that they are unlikely to aid epidemic spread in humans or provide a viable reservoir for the virus, at least in the immediate future. Our results are based on rabbits kept as pets, and further studies of rabbit farms and wild rabbits should be initiated to evaluate the transmission of SARS-CoV-2 in these different conditions.

## Data Availability

The data that supports the findings of this study are available from the corresponding author upon reasonable request.
